# Computationally Efficient Sources Location Method for Nested Array via Massive Virtual Difference Co-Array

**DOI:** 10.3390/s19091961

**Published:** 2019-04-26

**Authors:** Wei Wu, Yunfei Wang, Xiaofei Zhang, Jianfeng Li

**Affiliations:** 1 College of Electronic Information Engineering, Nanjing University of Aeronautics and Astronautics, Nanjing 211106, China; flyheart0307@nuaa.edu.cn (W.W.); wyf9612@126.com (Y.W.); zhangxiaofei@nuaa.edu.cn (X.Z.); 2 Institute of Manned Space System Engineering, China Academy of Space Technology, Beijing 100094, China; 3 Key Laboratory of Dynamic Cognitive System of Electromagnetic Spectrum Space (Nanjing University of Aeronautics and Astronautics), Ministry of Industry and Information Technology, Nanjing 211106, China; 4 College of computer and information, Hohai University, Nanjing 211100, China; 5 State Key Laboratory of Millimeter Waves, Southeast University, Nanjing 210096, China

**Keywords:** direction of arrival estimation, nested arrays, discrete Fourier transform, degrees of freedom

## Abstract

In this paper, we derive the discrete Fourier transform (DFT) method for direction of arrival (DOA) estimation by generating the massive virtual difference co-array with the nested array. By contrast with the spatial smoothing (SS) subspace-based methods for nested array, which halve the array aperture, the proposed method can take full advantage of the total array aperture. Since the conventional DFT method is a non-parametric method and is limited by Rayleigh threshold, we perform the phase rotation operation to obtain the fine DOA estimates. Owing to the full utilization of the array aperture and phase rotation operation, the proposed method can achieve better performance than SS subspace-based methods for far-field sources especially with massive virtual difference co-arrays which possess a large number of virtual sensors. Besides, as the fast Fourier transform (FFT) is attractive in practical implementation, the proposed method lowers the computational cost, as compared with the subspace-based methods. Numerical simulation results validate the superiority of the proposed method in both estimation performance and complexity.

## 1. Introduction

Direction of arrival (DOA) estimation is a significant subject in array signal processing and has various engineering applications such as radar, navigation and wireless communications [1,2,3,4,5,6,7,8]. In the past decades, various subspace-based algorithms, e.g., multiple signals classification (MUSIC) and estimation of signal parameters via rotational invariance techniques (ESPRIT), have been proposed for uniform linear arrays (ULAs) and have drawn considerable attention owing to their high resolution and performance [9,10,11]. Nevertheless, the inter-element spacing of ULAs is restricted to half wavelength to avoid spatial aliasing, which limits the array aperture and resultantly affects the estimation performance [12,13]. Besides, the number of resolvable sources of these conventional methods is less than the number of sensors. That is, an M-sensor array can only resolve M−1 sources at most. However, the problem of multiple sources detection is of great interest in many applications [14].

Recently, non-uniform arrays based on the concept of difference co-array have aroused wide attention due to the improvement of DOA estimation performance. In [15], the minimum redundancy array (MRA) is proposed to maximize the number of virtual sensors. Unfortunately, there is no simple closed-form expression for the array geometry as well as its achievable degrees of freedom (DOFs), which causes difficulties to array optimization and performance analysis. The coprime array [16,17,18,19,20,21,22,23,24], which consists of two uniform linear subarrays, can detect $O(M_{1}M_{2})$ sources using only $O(M_{1}\;+\;M_{2})$ physical sensors, where $M_{1}$ and $M_{2}$ denote the sensor number of the two subarrays. However, the coprime array, as well as its generalized configurations, have holes in its difference co-array, which limits the increase of the achievable DOFs. By contrast, a novel sparse array structure named the nested array [25,26,27,28,29,30,31] is proposed to obtain hole-free difference co-array, which means the nested array can achieve higher DOFs and, hence, achieve higher spatial resolution and better DOA estimation performance.

To take full advantage of the high DOFs offered by sparse arrays, a new approach which combines the spatial smoothing (SS) technique and the conventional MUSIC algorithm is proposed in [17]. The technique of SS [32] was first proposed to resolve the problem encountered in DOA estimation of the correlated signals. However, ref. [17] does not use the SS technique for decorrelating correlated sources as is the conventional way. Instead, the SS technique is utilized to build up the full rank of the observation matrix. However, the application of the SS technique halves the total DOFs obtained by difference co-array, hence the DOA estimation performance as well as the number of resolvable sources degrade. Another well-known algorithm that can solve DOA estimation problem of coherent signals is the maximum likelihood (ML) algorithm [33,34], which can obtain high estimation performance especially under low signal-to-noise ratio (SNR) and with small snapshots. Unfortunately, the ML algorithm involves multi-dimensional searches and hence brings prohibitively high computational complexity. In [35], the compressive sensing (CS) method [35,36,37,38,39] is applied to perform DOA estimation with sparse arrays by exploiting the sparsity property of the spatial signal spectrum. Unlike the SS technique, the compressive sensing method can make full use of DOFs offered by the virtual array, but at the cost of expensive complexity. Recently, a novel DOA estimation method based on discrete Fourier transform (DFT) is proposed in [40], which has a quite low complexity and needs merely one single snapshot, but is only suitable for massive ULA.

In this paper, we present the DFT method for DOA estimation by generating a massive virtual difference co-array with nested array. Different from the spatial smoothing (SS) subspace based methods for nested array which halves the array aperture, the proposed method takes full advantage of the total array aperture. Since the conventional DFT method is a non-parametric method and is limited by Rayleigh threshold, we perform the phase rotation operation to obtain the fine DOA estimates. Owing to the full utilization of the array aperture and phase rotation operation, the proposed method can achieve better performance than SS subspace-based methods for far-field sources especially with massive virtual difference co-arrays which possess a large number of virtual sensors. Since massive virtual sensors can be produced by a small number of physical sensors, the proposed algorithm can be quite practical in the scenarios where the array aperture and the number of antennas are limited, such as unmanned aerial vehicles (UAVs) and mobile devices, and it also has extensive applications in 5G communication, millimeter-wave communication and underwater acoustic positioning. Besides, as the fast Fourier transform (FFT) is attractive in practical implementation, the proposed method lowers the computational cost, as compared with the subspace-based methods. Due to the full utilization of the array aperture of the virtual difference co-array, the proposed method outperforms the commonly-used SS methods, e.g., SS-MUSIC and SS-ESPRIT [25] for far-field sources.

The main contribution of this paper are as follows.

(a) We extract the DFT method for the DOA estimation problem by constructing a massive virtual difference co-array with nested array. Besides, it is applicable to any sparse arrays which can generate a large virtual ULA, e.g., coprime arrays.
(b) The proposed method remarkably reduces the computational complexity since it applies FFT to obtain initial estimates and searches for fine estimates over a small refined region. Besides, it can avoid the process of eigenvalue decomposition (EVD) and has no need to know the number of sources in advance, which are all inevitable in the existing SS methods and CS methods.
(c) The proposed algorithm can utilize the full DOFs offered by the virtual array and hence increase the number of resolvable sources and improves estimation performance. Since the DFT method works well especially with large number of sensors, the proposed method can obtain pretty high accuracy by using massive virtual difference co-arrays generated by nested array.

The rest of this paper is organized as follows. The nested array geometry and data model are presented in Section 2. The combination of the DFT method and the nested array is presented in Section 3 and the analysis of the algorithm performance is provided in Section 4. Simulation results are provided in Section 5 and Section 6 concludes the paper.

Notations: throughout this paper, we use uppercase (lowercase) bold characters to denote matrices (vectors). ${\left(\cdot \right)}^{T}$, ${\left(\cdot \right)}^{*}$, ${\left(\cdot \right)}^{H}$ represent the transpose, conjugate and transpose conjugate operators. E{·} represents the expectation operator. vec{A} stacks the columns of the matrix A to generate a long vector, and the symbol ∘ denotes the Khatri–Rao product between two matrices.

## 2. Signal Model of Nested Array

**Figure 1:**
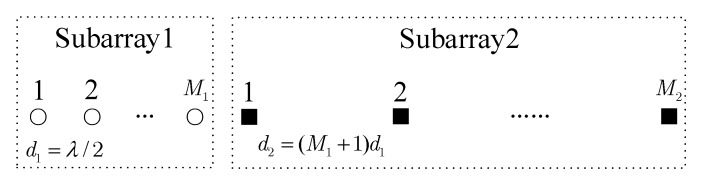
A two-level nested array geometry.

Consider a two-level nested array [25] as shown in Figure 1. The inter-element spacing of the first subarray with $M_{1}$ sensors is $d_{1}=\lambda /2$ while the second subarrays with $M_{2}$ sensors is $d_{2}=(M_{1}+1)d_{1}$, where λ denotes the wavelength. The spacing between the two subarrays is $d_{1}$. The total number of sensors is $M=M_{1}+M_{2}$. The locations of the sensors in the nested array are in the set $L_{s}=\{md_{1}|m=0,1,\cdots ,M_{1}-1\}\cup \{M_{1}d_{1}+nd_{2}|n=0,1,\cdots ,M_{2}-1\}$. Without loss of generality, we assume that M is an even integer. According to [25], we take the optimal two-level nested array into account with $M_{1}=M_{2}=M/2$.

Assume that there are *K* far-field uncorrelated narrowband signals impinging on the nested array from different angles located at $\theta_{k}(k=1,2\cdots ,K)$. The received signal of the nested array can be represented as [33]

(1) $$x(t)\;=\;As(t)\;+\;n(t)\;=\;\sum_{k=1}^{K}a(\theta_{k})s(t)+n(t)$$

where $A=[a(\theta_{1}),a(\theta_{2}),\cdots ,a(\theta_{K})]\in {\mathbb{C}}^{M\times K}$ is the manifold matrix and $a(\theta_{k})=[1,e^{j2\pi l_{2}\sin\theta_{k}/\lambda },\cdots ,$ $e^{j2\pi l_{M}\sin\theta_{k}/\lambda }{]}^{T}\in {\mathbb{C}}^{M\times 1}$ is steering vector, where $l_{m}\in L_{s}$(m=1,⋯,M) denotes the position of sensors, $s(t)={\left[s_{1}(t),\cdots ,s_{K}(t)\right]}^{T}\in {\mathbb{C}}^{K\times 1}$ is the signal vector and n(t) stands for the additive white Gaussian noise with mean zero and variance $\sigma_{n}^{2}$, which is uncorrelated with signals. Here, we assume the number of sources K is known.

The covariance matrix of the signal can be represented as [25]:

(2) $$R=E\left\{x(t)x^{H}(t)\right\}=\sum_{k=1}^{K}P_{k}a(\theta_{k})a^{H}(\theta_{k})+\sigma_{n}^{2}I_{M}$$

where $P_{k}$ is the average power of the k-th signal and $I_{M}\in {\mathbb{C}}^{M\times M}$ denotes the identity matrix. In practice, the covariance matrix of the signal with a finite number of snapshots [41] is:

(3) $$\hat{R}=\sum_{l=1}^{L}x(l)x^{H}(l)/L$$

where l=1,2,⋯,L and L stands for the number of snapshots.

## 3. Proposed Method for Direction of Arrival (DOA) Estimation

### 3.1. Vectorization of the Covariance Matrix

According to [42], the covariance matrix of the signal with a finite number of snapshots can be vectorized as:

(4) $$y=vec\left(\hat{R}\right)=\left(A^{*}\circ A\right)p+\sigma_{n}^{2}U=A_{e}p+\sigma_{n}^{2}U$$

where $p={\left[P_{1},P_{2},\cdots ,P_{K}\right]}^{T}$, $U=vec\left\{I_{M}\right\}$, $A_{e}=A^{*}\circ A$ and $I_{M}\in {\mathbb{C}}^{M\times M}$ is an identity matrix. Note that the vector **y** in (4) can be treated as an extended received signal observing with a much longer virtual ULA. It has been presented in [25] that a $(M^{2}+2M-2)/2\times K$ matrix $A_{v}$ can be obtained by removing the repeated rows from $A_{e}$ and the positions of sensors in the virtual consecutive ULA are from $(-M^{2}/4-M/2+1)d_{1}$ to $(M^{2}/4+M/2-1)d_{1}$. The total number of sensors of the virtual ULA is $N=(M^{2}+2M-2)/2$. The virtual observation vector is given by:

(5) $$y_{v}=A_{v}p+\sigma_{n}^{2}u$$

where $A_{v}=[a_{v}(\theta_{1}),\cdots ,a_{v}(\theta_{K})]\in {\mathbb{C}}^{N\times K}$ is the direction matrix for the virtual array, whose (n,k)-th element is $e^{j2\pi n\sin\theta_{k}/\lambda }$(n=−(N−1)/2,⋯,(N−1)/2). p can be seen as the signal vector, and $u\in {\mathbb{R}}^{N\times 1}$ is a vector with a one at the ((1+N)/2)-th position and the other elements are all zeros.

In [25], by applying the SS technique to the observation vector $y_{v}$, an [(N+1)/2]×[(N+1)/2] matrix $R_{ss}$ as the SS covariance matrix can be achieved. However, almost half the aperture of the virtual ULA is dissipated. To fully utilize the aperture provided by a nested array, we apply the DFT method [40] to $y_{v}$.

### 3.2. Coarse Initial Estimation

Define the normalized DFT matrix $F\in {\mathbb{C}}^{N\times N}$ as:

(6) $$F=\frac{1}{\sqrt{N}}\left[\begin{array}{cccc} W_{N}^{1} & W_{N}^{2} & \cdots & W_{N}^{N}\\ W_{N}^{2} & W_{N}^{4} & \cdots & W_{N}^{2N}\\ \vdots & \vdots & & \vdots \\ W_{N}^{N} & W_{N}^{2N} & \cdots & W_{N}^{N^{2}} \end{array}\right]$$

where the (p,q)-th element of F is $W_{N}^{pq}=e^{-j2\pi pq/N}$. Then the DFT of the virtual steering vector is:

(7) $${\tilde{a}}_{v}(\theta_{k})=Fa_{v}(\theta_{k})$$

whose q-th element is [40]:

(8) $$\begin{array}{l} {\left[{\tilde{a}}_{v}(\theta_{k})\right]}_{p}=\frac{1}{\sqrt{N}}\sum_{i=0}^{N-1}e^{-j(\frac{2\pi }{N}p-\pi \sin\theta_{k})i}\\ \;=\frac{1}{\sqrt{N}}\frac{\sin\left[\frac{N}{2}\left(\frac{2\pi }{N}p-\pi \sin\theta_{k}\right)\right]}{\sin\left[\frac{1}{2}\left(\frac{2\pi }{N}p-\pi \sin\theta_{k}\right)\right]}e^{-j\frac{N-1}{2}\left[\frac{2\pi }{N}p-\pi \sin\theta_{k}\right]} \end{array}$$

According to [40], if the number of sensors in the virtual consecutive ULA is infinite, i.e., N→∞, as:

(9) $$\underset{N\to \infty }{\lim}\left|{\left[{\tilde{a}}_{v}(\theta_{k})\right]}_{p}\right|=\sqrt{N}\delta (\frac{2\pi }{N}p-\pi \sin\theta_{k})$$

where δ(⋅) is the delta function, there always exists an integer $p_{k}=N\sin\theta_{k}/2$ that makes ${\left[{\tilde{a}}_{v}(\theta_{k})\right]}_{p_{k}}=\sqrt{N}$ and the others are all zeros. Subsequently, the estimate of $\theta_{k}$(k=1,2,⋯,K) can be obtained from ${\tilde{a}}_{v}(\theta_{k})$. However, in practice, the number of sensors of an array is finite even in a massive multiple-input multiple-output (MIMO) system [43]. Then, the power of the $round\{N\sin\theta_{k}/2\}$-th DFT point will not be concentrated at this point, which will depress the accuracy but still can be taken as initial estimates.

Therefore, the DFT of the observation vector can be obtained by $z=Fy_{v}$ and the p-th element is presented as:

(10) $${\left[z\right]}_{p}=\sum_{k=1}^{K}{\left[{\tilde{a}}_{v}(\theta_{k})\right]}_{p}P_{k}+\sigma_{n}^{2}{\left[Fu\right]}_{p}$$

Accordingly, **z** has K peaks and we can search the spectrum of **z** for K largest values whose indices are denoted as $p_{k}^{ini}$, and obtain their corresponding initial DOA estimates, denoted as:

(11) $$\theta_{k}^{ini}=\sin^{-1}(2p_{k}^{ini}/N),\;k=1,2,\cdots ,K$$

### 3.3. Fine Estimation

The resolution and estimation accuracy of the DFT method is limited by the Rayleigh limit. However, we will show that the accuracy of the DFT method can be improved with the use of the total array aperture and phase rotation operation.

To decrease the estimation error caused by a finite number of sensors, a phase rotation operation is conducted. The phase rotation matrix is defined as [40]:

(12) $$\Phi (\eta )=diag\left\{1,e^{j\eta },\cdots ,e^{j(N-1)\eta }\right\}$$

where η∈(−π/N,π/N) is related to the phase shifter.

The steering vector corrected by rotation matrix can be achieved as:

(13) $${\tilde{a}}_{v}^{ro}(\theta_{k})=F\Phi (\eta ){\tilde{a}}_{v}(\theta_{k})$$

where the p-th element can be denoted as:

(14) $${\left[{\tilde{a}}_{v}^{ro}(\theta_{k})\right]}_{p}=\frac{1}{\sqrt{N}}\frac{\sin\left[\frac{N}{2}\left(\frac{2\pi }{N}p-\eta -\pi \sin\theta_{k}\right)\right]}{\sin\left[\frac{1}{2}\left(\frac{2\pi }{N}p-\eta -\pi \sin\theta_{k}\right)\right]}e^{-j\frac{N-1}{2}\left[\frac{2\pi }{N}p-\eta -\pi \sin\theta_{k}\right]}$$

Similarly, there must exists a corresponding phase shifter between (−π/N,π/N), referred to as the optimal phase shifter $\eta_{k}$, which makes:

(15) $$\frac{2\pi }{N}p_{k}^{ini}-\eta_{k}=\pi \sin\theta_{k}$$

In this case, the rotated steering vector ${\tilde{a}}_{v}^{ro}(\theta_{k})$ would have only one non-zero element. Then the fine estimate of $\theta_{k}$ (k=1,2,⋯,K) can be obtained as:

(16) $$\theta_{k}^{fine}=sin^{-1}(\frac{2p_{k}^{ini}}{N}-\frac{\eta_{k}}{\pi })$$

We can construct the cost function:

(17) $$\eta_{k}=\arg\underset{\eta \in (-\frac{\pi }{N},\frac{\pi }{N})}{\max}{\left\|f_{p_{k}^{ini}}^{H}\Phi (\eta )y_{v}\right\|}^{2}$$

where $f_{p_{k}^{ini}}$ denotes the $p_{k}^{ini}$-th column of the DFT matrix F.

By searching η over a small sector (−π/N,π/N) and the optimal phase shifter $\eta_{k}$ can be obtained by solving the K peaks of $z^{ro}=F\Phi y_{v}$. Owing to the phase shifter operation, the power of DFT spectrum is centralized around the theoretical DOA of the signal and hence the performance of the DOA estimation can be greatly improved.

**Remark** **1.** *In practice, we usually use FFT rather than DFT to accelerate the calculation and in this case, we need to employ the zero-padding method [36] if the number of sensors of the virtual ULA is not an integer power of 2.*

**Remark** **2.** *According to the above derivation of the proposed algorithm, it has no need to know the number of sources in advance, which is attractive in practical implementation.*

**Remark** **3.** *The DFT based method is also suitable for other arrays that can be transformed to a virtual ULA, such as the coprime array [16].*

### 3.4. Detailed Steps

The detailed steps of the proposed method are as follows:

Step 1 Compute the covariance matrix and get the observation vector $y_{v}$ in (5);
Step 2 Compute the DFT of $y_{v}$ and search the spectrum of z for K values to obtain $\theta_{k}^{ini}$ (k=1,2,⋯,K);
Step 3 Compute $z^{ro}$ and search η over (−π/N,π/N) to obtain the optimal phase shifter $\eta_{k}$ (k=1,2,⋯,K);
Step 4 Compute fine DOA estimates according to (16).

**Remark** **4.** *Our method is designed for far-field sources coming from different directions. Our method fails to resolve sources with the same directions but different ranges. Such sources can be resolved in the near-field cases [44,45,46].*

## 4. Performance Analysis

### 4.1. Computational Complexity

In this subsection, we mainly discuss the complex multiplication operation. Specifically, computing covariance matrix of the received signal needs the complexity of $O(M^{2}L)$. To accelerate computation, we apply FFT with the complexity of O(NlogN). Besides, the process of peak locating and shifted phase searching needs O(N) and O(2πK/Δ) respectively, where Δ denotes the search grid. To sum up, the total complexity of the proposed method is $O(M^{2}L+N\log N+N$ +2πK/Δ). It should be noted that small Δ leads to high accuracy but high computational complexity as well. However, for massive nested arrays which can produce extremely large number of virtual sensors, high accuracy and low complexity can be obtained even with a large search grid Δ.

**Figure 2:**
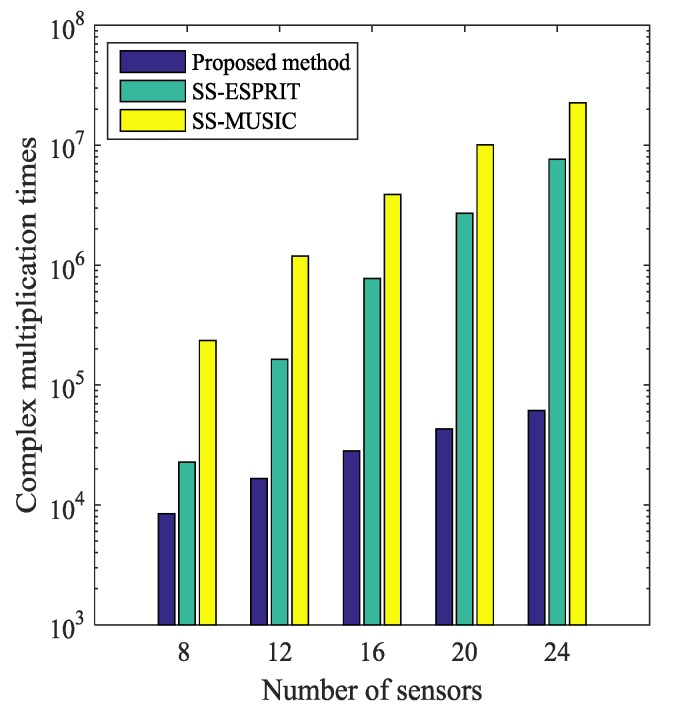
Comparison of complexity versus number of sensors.

Specific complexities of the proposed method along with the SS-ESPRIT [25] and the SS-MUSIC [25] are listed in Table 1 for clarity. The comparison of the computational complexity versus number of sensors is illustrated in Figure 2, where K=3, L=100 and the search grid Δ  =  0.0001  rad. As the proposed method does not need to perform EVD and searches over a small sector, it shows clearly that its complexity is much lower than the SS methods.

### 4.2. Resolution and DOF

The resolution of the DFT method is limited by the Rayleigh limit, it cannot resolve sources between two adjacent peaks of the spectrum. Two close sources at $\theta_{1}$ and $\theta_{2}$ can be resolved only when $|\sin\theta_{1}\;-\;\sin\theta_{2}|\;\geq \;4/N$ holds, where N is the number of sensors of the virtual ULA.

**Figure 3:**
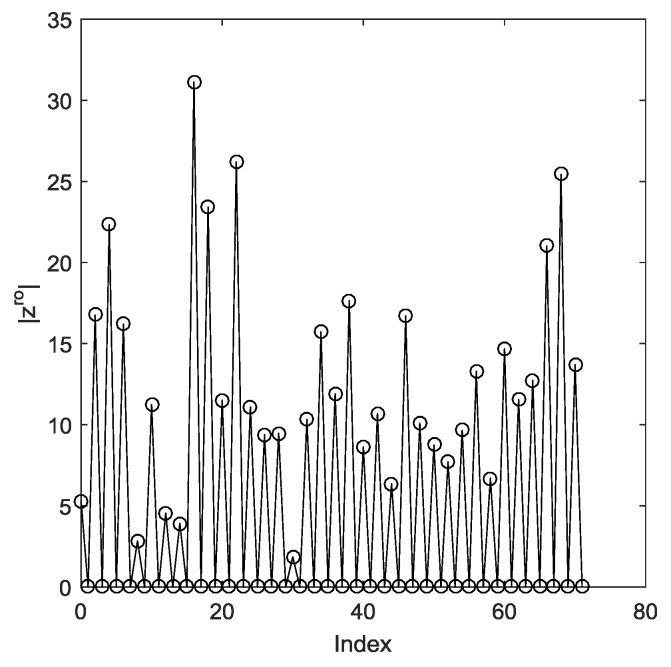
Maximum number of peaks in the positive axis.

The maximum number of sources that the DFT method can resolve is ⌊N/2⌋. Figure 3 shows the maximum peaks in the positive axis without considering the noise, where the DOAs $\theta_{p}=\sin^{-1}(4p/N),p=0,1,\cdots ,\left\lfloor N/4\right\rfloor$ correspond to the even indices of the peaks and M=16,N=143. We can see that all 36 peaks can be located and there are 35 peaks in the negative axis, all 71 peaks can be located for specific DOAs (correspond to the odd or even integer indices exactly).

### 4.3. Advantages

- The proposed method outperforms SS-ESPRIT and SS-MUSIC [25] for distant sources DOA estimation with nested arrays as it involves the full aperture of the virtual ULA and a phase rotation operation is applied for fine estimation.
- The proposed method is computationally efficient since it applies DFT (FFT) and searches for peaks over a small sector. Besides, it can avoid performing EVD operation, which is time-consuming and inevitable in SS methods.
- The proposed algorithm has no need to know the number of sources in advance, which is practical and attractive in the realistic scenario.

## 5. Simulation Results

In the simulation section, we only consider the two-level optimal nested array, i.e., two subarrays have the same number of sensors. The root mean square error (RMSE) is used as the performance comparison metric, which is defined as:

(18) $$\mathrm{RMSE}=\sqrt{\sum_{q=1}^{Q}\sum_{k=1}^{K}{\left({\hat{\theta }}_{k,q}-\theta_{k}\right)}^{2}/QK}$$

where Q is the number of Monte Carlo simulations, ${\hat{\theta }}_{k,q}$ is the estimate of the q-th trial for the k-th theoretical angle $\theta_{k}$. And in this paper, we set Q=1000.

**Figure 4:**
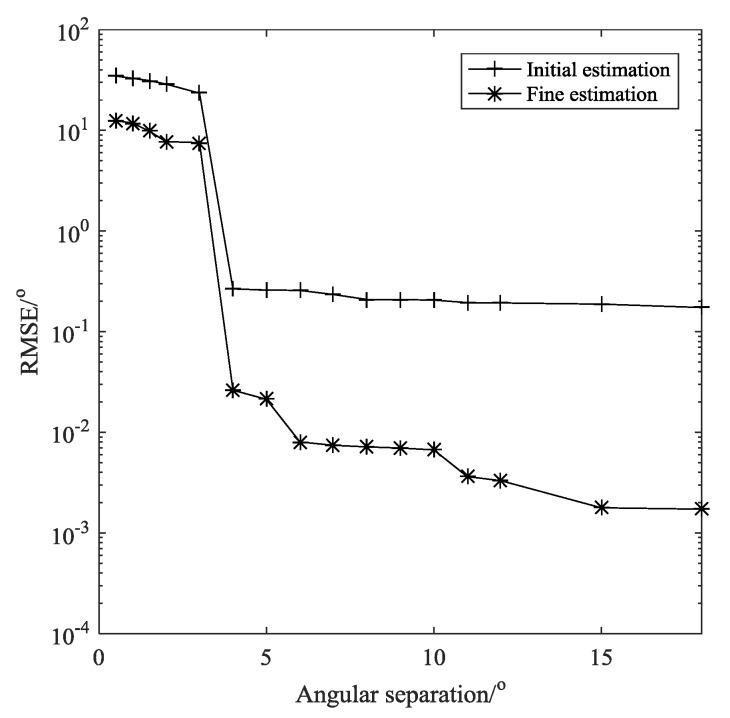
Root mean square error (RMSE) versus angular separation.

**Example** **1.** *The DFT based method is a non-parametric method and its resolution is restricted by the Rayleigh limit. Figure 4 shows the RMSE of the proposed method with the increase of the angular separation of two sources, where*M=  16, L = 200, SNR = 20 dB, N=143*, and the search grid for fine estimation is*Δ=0.0001 rad*. As mentioned above, the resolution is determined by the aperture of virtual array as*$\sin^{-1}(4/N)$*. In this case, the angular resolution is about*$1.6^{{}^{\circ}}$*. It is depicted clearly in Figure 4 that, when the separation is less than*$2^{{}^{\circ}}$*, the proposed algorithm has difficulty to resolve the two sources. But when sources get far, the estimation accuracy improves significantly owing to the phase rotation operation. Considering the large virtual aperture the massive nested arrays can produce, the proposed method can obtain a rather high resolution.*

**Figure 5:**
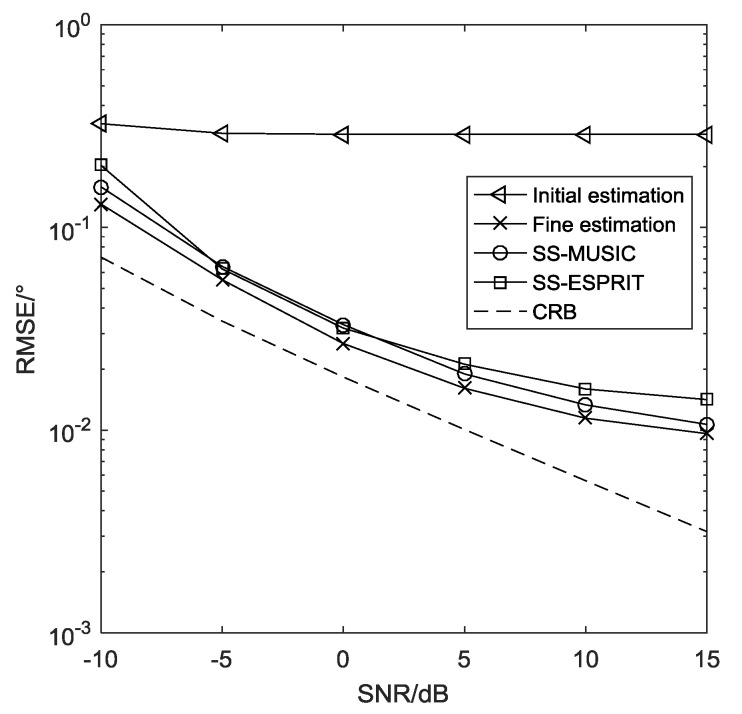
RMSE comparison of different methods.

**Example** **2.** *The RMSE comparison of the proposed DFT method and SS methods [25] (SS-MUSIC and SS-ESPRIT) for three distant sources*$\theta =[10^{\circ },30^{\circ },50^{\circ }]$*is given in Figure 5, where*M=16, L=200*, and the search grid is set to be*Δ=0.0001 rad*for both the proposed DFT algorithm and the SS-MUSIC algorithm. Moreover, the Cramer-Rao Bound (CRB) [47,48] of the nested array is also provided in Figure 5 to evaluate the theoretical performance of the proposed method. It is shown clearly that the DFT method outperforms the others as it can utilize the whole virtual consecutive array, while the SS methods sacrifice half of the array aperture to perform spatial smoothing technique.*

**Figure 6:**
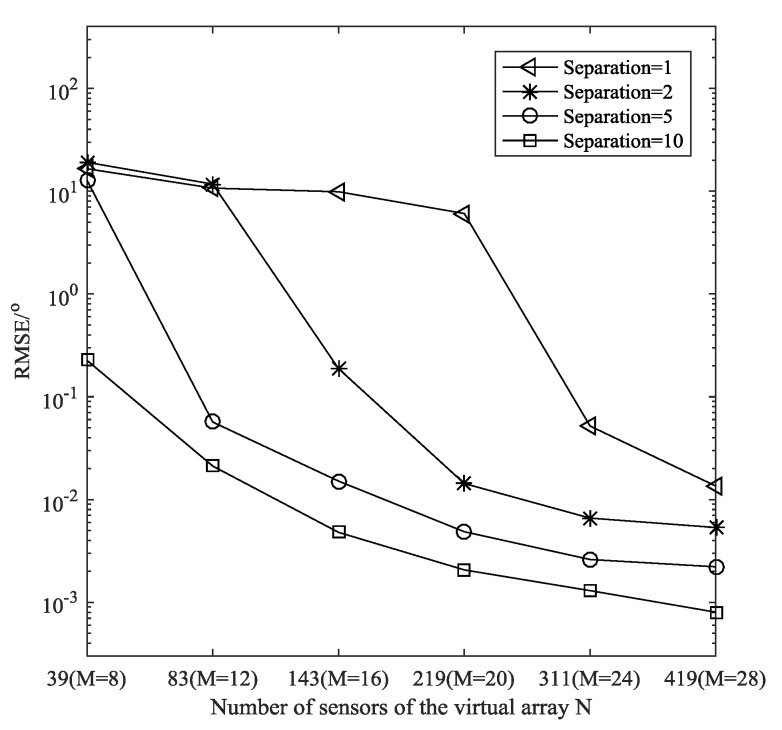
Estimation performance with different angular separation.

**Example** **3.** *In this example, we compare the estimation accuracy versus the number of sensors for different angular separations, where*L=500, SNR=20 dB, Δ = 0.0001 rad*. As shown in Figure 6, more sensors are needed for closer sources. To resolve sources with separation*$1^{{}^{\circ}}$, $2^{{}^{\circ}}$, $5^{{}^{\circ}}$, $10^{{}^{\circ}}$*, the two-level nested array needs about 24, 20, 12 and 8 sensors respectively.*

**Figure 7:**
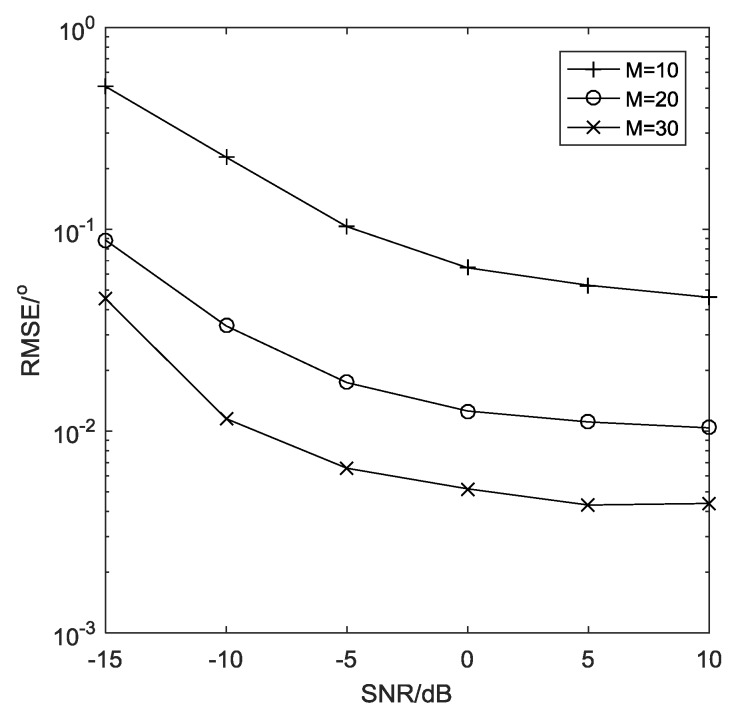
Estimation performance with different M.

**Example** **4.** *The estimation performance of the proposed algorithm with different number of sensors is illustrated in Figure 7, where the snapshots is* L=1000 *and the search grid is* Δ=0.0001 rad *. Obviously, the performance of the proposed algorithm improves a lot as the number of sensors increases. Since the massive nested array can produce an extremely large number of virtual sensors, the proposed algorithm can obtain high angular resolution as well as high estimation performance at the same time.*

**Figure 8:**
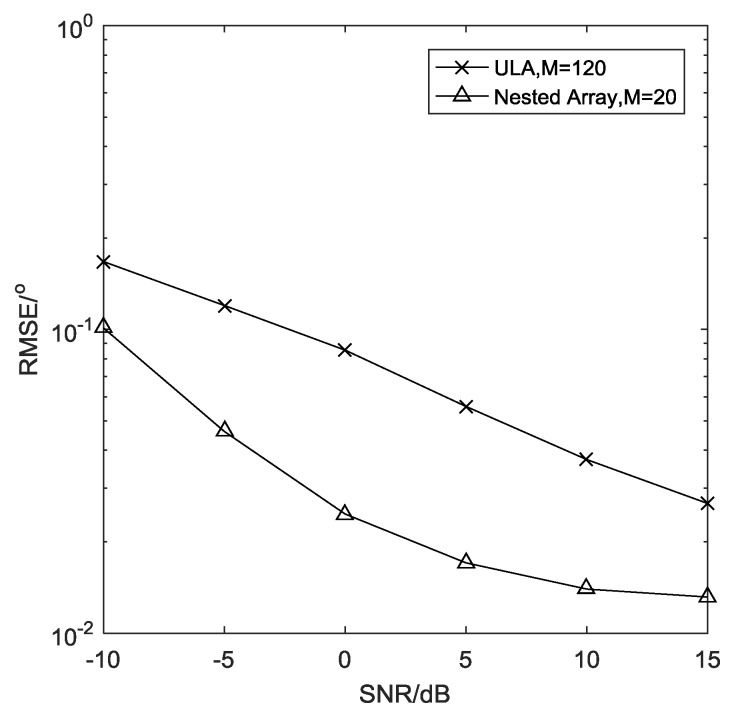
RMSE comparison of uniform linear array (ULA) and nested array.

**Example** **5.** *In this example, we compare the estimation performance of ULA and nested array, where*L=500, Δ = 0.0001 rad*, the total number of physical sensors is*M = 120*for ULA, and*M = 20*for the nested array. It is shown explicitly in Figure 8 that owing to the large aperture of its virtual array, the nested array can achieve better estimation performance even with a much smaller number of physical sensors.*

**Figure 9:**
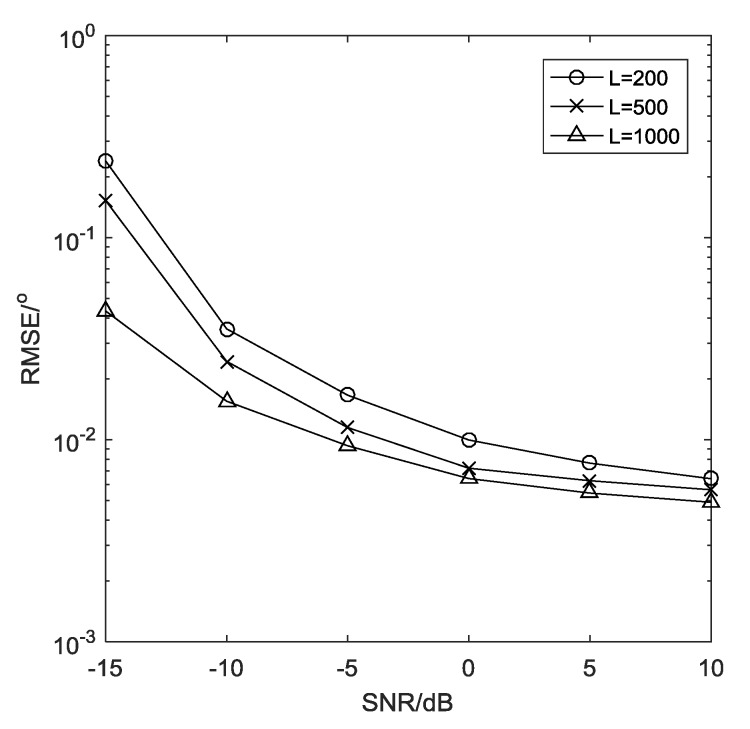
Estimation performance with different L.

**Example** **6.** *Figure 9 compares the estimation performance with different number of snapshots, where*M = 26, SNR=20 dB, Δ = 0.0001 rad*. It shows clearly that the performance of angle estimation becomes better with the number of snapshots increasing.*

## 6. Conclusions

In this paper, we derive the DFT method for DOA estimation with a massive virtual difference co-array generated by nested array. By contrast with the SS subspace-based methods for a nested array which halves the array aperture, the proposed method takes full advantage of the total array aperture. Moreover, since the conventional DFT method is a non-parametric method and is limited by Rayleigh threshold, we conduct the phase rotation operation to achieve fine DOA estimates. Specifically, due to the full utilization of the array aperture and phase rotation operation, the proposed method can attain better performance than SS subspace-based methods for far-field sources especially with a massive virtual difference co-array which consists of a large number of virtual sensors. Besides, as the FFT is attractive in practical implementation, the proposed method lowers the computational cost, as compared with the subspace-based methods. Due to the full utilization of the array aperture of the virtual difference co-array, the proposed method outperforms the commonly-used SS methods for far-field sources.

## Figures and Tables

**Table 1:** Complexity of different methods.

Method	Complexity
Proposed	$O(M^{2}L+N\log N+N+2\pi K/\Delta )$
SS-ESPRIT	$O(M^{2}L+2{\left((N+1)/2\right)}^{3}+(N-1)K^{2}+3K^{3})$
SS-MUSIC	$O(M^{2}L+2{\left((N+1)/2\right)}^{3}+180N(N/2-K)/2)/\Delta$
